# Postconcussion Symptoms in Patients with Injury-Related Chronic Pain

**DOI:** 10.1155/2012/528265

**Published:** 2012-05-22

**Authors:** Britt Marie Stålnacke

**Affiliations:** Department of Community Medicine and Rehabilitation, Umeå University Hospital, Umeå University, Building 9A, 90185 Umeå, Sweden

## Abstract

*Background*. Postconcussion symptoms (PCSs)—such as fatigue, headache, irritability, dizziness, and impaired memory—are commonly reported in patients who have mild traumatic brain injuries (MTBIs). Evaluation of PCS after MTBI is proposed to have a diagnostic value although it is unclear whether PCS are specific to MTBI. After whiplash injuries, patients most often complain of headaches and neck pain; the other PCS are not as closely evaluated. In patients with chronic pain because of other injuries, the presence of PCS is unclear. This study aimed to describe the frequency of PCS in patients with injury-related pain and to examine the relationships between PCS, pain, and psychological factors. *Methods*. This study collected data using questionnaires addressing PCS (Rivermead Postconcussion Questionnaire, RPQ), pain intensity (Visual Analogue Scale), depression, anxiety (Hospital, Anxiety, and Depression Scale), and posttraumatic stress (Impact of Event Scale). *Results*. Fatigue (90.7%), sleep disturbance (84.9%), headache (73.5%), poor concentration (88.2%), and poor memory (67.1%) were some of the most commonly reported PCS. Significant relationships were found between PCS and posttraumatic stress, depression, and anxiety. *Conclusion*. To optimize treatment, it is important to assess each patient's PCS, the mechanism of injury, and factors such as posttraumatic stress and depression.

## 1. Introduction

The great majority (80–90%) of traumatic brain injuries is classified as mild traumatic brain injury (MTBI)/concussion [1, 2]. These injuries are a matter for general concern because of their potential long-term consequences—persistent post-concussion symptoms (PCSs). PCS include headache, fatigue, dizziness, and impaired memory. These symptoms can affect both work and leisure time [3–5]. Although the natural course of recovery after MTBI consists of restitution of many symptoms within three months after injury [6], a significant number of persons report PCSs that last for many months to even years after injury. Evaluation of these symptoms after MTBI can have diagnostic value. For patients with longterm symptoms, a diagnosis of post-concussion syndrome is sometimes used. According to the ICD10 criteria, at least three symptoms, which may include headache, dizziness, fatigue, depression, irritability, difficulty in concentration, and memory problems, are required for a diagnosis of postconcussion syndrome [7]. The DSM IV criteria for postconcussion disorder include evidence of three or more of these symptoms present for at least three months combined with signs of impaired cognitive function and social disability [8].

Whether PCS are specific to MTBI is unclear since symptoms commonly reported after MTBI have been reported in the general population and by patients with chronic pain. Some of the factors that have been found to contribute to the persistence of symptoms are depression and posttraumatic stress [9, 10].

Chronic pain is an acute and/or intermittent pain that persists more than three months [11], and the great majority of chronic pain is musculoskeletal pain. Apart from individual suffering, chronic pain is costly for society. Some of the most common causes of musculoskeletal pain are injuries related to traffic accidents, falls, and sports. In western countries, whiplash as the result of traffic accidents has a very high annual incidence: 1.0 to 3.2/1000 per year [12]. Whiplash is an acceleration-deceleration mechanism of energy transferred to the neck that may result in soft tissue injury/distortion of the neck. Most studies of the long-term outcome of whiplash-associated disorder (WAD) have focused on neck pain and headache since these are the dominating complaints, while other PCSs are less investigated. Cognitive symptoms such as memory and concentration difficulties have been reported in patients with whiplash injuries, but the relevance of these symptoms has not been fully examined. Although some studies have shown neuropsychological dysfunction in persons with long-term cognitive symptoms after whiplash injury [13, 14], other studies have suggested neurotic development or preexistent stress as the underlying cause [15].

Although most studies suggest that female gender is a potential prognostic factor related to poor recovery both after MTBI [16] and whiplash injuries [17], some studies fail to show any gender differences regarding chronic symptoms as the result of MTBI and whiplash injuries [6, 18]. For MTBI patients with PCS, some studies have divided the symptoms into three subgroups: emotional, cognitive, and physical [19, 20]. For example, when comparing patients with chronic pain and patients with MTBI, Smith-Seemlier et al. found that cognitive difficulties were more often reported by the MTBI patients [19]. No group differences were found for total scores of postconcussion symptoms. Since their study focused on chronic pain patients regardless of the cause of pain, it may be of interest to study symptoms in patients with injury-related chronic pain.

The aims of the present study were (i) to describe the frequency of postconcussion symptoms in patients with injury-related chronic pain, (ii) to study the relationships between postconcussion symptoms and pain intensity, posttraumatic stress, and depression, (iii) and to examine gender differences regarding these variables.

## 2. Patients and Methods

A cross-sectional study design was used to study patients with chronic pain caused by an injury and referred by regional general practitioners to the Pain Rehabilitation Clinic at the Umeå University Hospital (Umeå, Sweden). The study included 86 patients—59 women and 27 men (aged 41.1 ± 10.3 years). The participants suffered from pain caused by falls (14.0%), whiplash injuries (44.4%), other nonwhiplash traffic injuries sustained as the result of bicycle and motorcycle accidents (8.1%), horseback riding (8.1%), sports (1.2%), assaults (5.8%), and other injuries such as work-related injuries (18.4%). For all patients, the time between injury and assessment was more than one year.

### 2.1. Assessments

During assessment in the clinic, the patients answered a set of questionnaires. Information about each participant's trauma history was collected from hospital records. Symptoms (pain intensity and whiplash-related symptoms) were assessed using the Visual Analogue Scale [21], the Rivermead Postconcussion Symptoms Questionnaire [22], the Impact of Event Scale [23], and the Hospital Anxiety and Depression Scale [24].

### 2.2. The Visual Analogue Scale

Pain intensity was rated using the Visual Analogue Scale (VAS) [21]. The scale consists of a 100 mm straight line with defined end points (“no pain” and “worst pain imaginable”) on which the patients were asked to mark their experienced pain (results in mm). The VAS is considered to have a high degree of reliability and validity.

### 2.3. Rivermead Postconcussion Symptoms Questionnaire

The Rivermead Postconcussion Symptoms Questionnaire (RPQ) [22] is a validated instrument that was used to assess the frequency and severity of 16 symptoms that are commonly encountered postconcussion symptoms. The RPQ asks patients to rate the extent to which their symptoms (compared with their premorbid levels) have become more problematic over the previous 24 hours. The RPQ uses a rating scale with values 0–4, from no problem at all to a severe problem. A total symptom score can be calculated as a sum of all scores (possible score 0–72) [22].

### 2.4. The Impact of Event Scale

The Impact of Event Scale (IES) is a widely used self-report scale [23]. It is a valid measure of posttraumatic stress reactions and has been suggested as a screening tool for posttraumatic stress disorder (PTSD). The IES comprises 15 statements: seven questions about intrusive symptoms and eight questions about avoidance symptoms. The patients answer the questionnaire regarding their symptoms during the last week. The total score, which varies between 0 and 80, is divided into four stress reaction grades: subclinical (0–8), mild (9–25), moderate (26–43), and severe (44–80) [23].

### 2.5. HAD

To measure anxiety and depression, the Hospital Anxiety and Depression Scale (HAD) was developed and validated on nonpsychiatric medical patients [24]. The questionnaire comprises of 14 items divided in two parts, for rating of depression and anxiety. Each item has a four response category ranging between 0 and 3. The scale ranges between 0 and 21 for both depression and anxiety. According to Zigmond and Snaith, the cut-off level for possible cases of anxiety disorder and depression is a score ≥ 8 on each subscale [24].

### 2.6. Statistical Analyses

All statistical analyses were performed with SPSS for Windows (version 19.0). Data are reported as means + standard deviations unless indicated otherwise. Comparisons of populations were made using the Mann-Whitney *U* test. Pearson's correlation coefficient was calculated for the analysis of bivariate correlations. Statistical significant level was set at 0.05.

The study was approved by the ethics committee of Umeå University.

## 3. Results

### 3.1. Pain Intensity

For all patients, the pain intensity on the VAS was 65.8 ± 20.2 mm. No statistically significant difference was found between women (65.5 ± 21.1 mm) and men (66.8 ± 18.2 mm).

### 3.2. Postconcussion Symptoms

The most common PCSs reported were fatigue, sleep disturbance, and poor concentration (Table 1). The most common symptoms rated as a problem were fatigue, sleep disturbance, and headache (Table 2). Statistically significant differences between women and men were only found for the symptoms sleep disturbance, feeling frustrated, blurred vision, and taking longer to think (Table 1). No statistically significant difference between men and women was found for the total RPQ score.

### 3.3. Posttraumatic Stress

The total score of the IES for all patients was 19.3 ± 15.0, and the scores for the subscales were avoidance 9.8 ± 9.1, and intrusion 9.5 ± 7.3. Mild level of posttraumatic stress was reported by 37.2%, moderate stress by 22.1%, and severe stress by 8.1%. No statistically significant differences between men and women were found with respect to total IES (men: 19.7 ± 13.3; women: 19.1 ± 15.8), avoidance (men: 10.2 ± 8.8; women: 9.6 ± 9.3), and intrusion (men: 9.5 ± 5.9; women: 9.5 ± 7.8).

### 3.4. Depression

Depression scores on the HAD for all patients were 6.9 ± 4.4 mm (women: 7.4 ± 4.3; men: 5.9 ± 3.9). A significantly higher proportion of women (47.5%) reported possible-probable depression (HAD score ≥ 8) compared to men (22.2%) (*P* = 0.038).

### 3.5. Correlations

Total score of RPQ was significantly correlated to posttraumatic stress (*r* = 0.375, *P* < 0.001), HAD anxiety (*r* = 0.455, *P* < 0.001), and HAD depression (*r* = 0.560, *P* < 0.001). No significant correlation was found between RPQ and VAS (*r* = 0.150, *P* = 0.183).

## 4. Discussion

The present study shows that patients with injury-related pain often reported postconcussion symptoms several years after injury. Although more women than men participated in the study, few differences between genders were found. A significant relationship was found between postconcussion symptoms and posttraumatic stress and between postconcussion symptoms and depression and anxiety.

As whiplash is reported as the most common traffic injury, it was not surprising that most patients related their chronic pain condition to a previous whiplash trauma. Previous studies have used several different constructions of questionnaires to ask about postconcussion-like symptoms after whiplash [14, 15]. Neck pain and headache are the most often reported complaints after whiplash injury, but other symptoms such as dizziness and visual impairments have also been reported [15]. In comparison with a previous follow-up study that also used the RPQ to examine persons five years after whiplash trauma [25], the proportion of the separate symptoms, except for poor concentration, was clearly higher in the present study. The differences may be due to different study populations. Patients in the present study represent a selected group of patients with chronic pain who exhibited severe consequences after their injury and who were referred to a specialist clinic.

The frequencies of symptoms were high and clearly higher than that for MTBI patients who reported on the RPQ from our hospital one year after the trauma [5]. The most common problematic symptoms were headache, fatigue, and sleep disturbance. In a previous study from Canada of MTBI-patients that also used the RPQ, these symptoms were also the most frequently cited both ten days and six weeks after injury [26]. In patients with MTBI and chronic pain, sleep dysfunction is common. Sleep dysfunction is important to assess since sleep dysfunction and fatigue have been shown to aggravate pain and other symptoms [27]. The frequency of cognitive symptoms in the present study was surprisingly high; more than half of the patients described memory and concentration difficulties. Cognitive difficulties are often related to neuropsychological impairments after MTBI, but not all patients are investigated using neuropsychological tests. These patients are screened using self-reported symptoms of memory and concentration dysfunction [6]. In addition, pain has been associated with worse cognitive functioning in persons with a traumatic brain injury (TBI). Pain, posttraumatic stress, and depression all could cause prolonged cognitive impairment after MTBI [15]. In the present study, these factors were also significantly correlated to post-concussion symptoms.

Some limitations of this study should be noted. General practitioners referred patients to a pain rehabilitation clinic because they reported injury-related chronic pain due to an injury sustained more than twelve months since the referral. Thus the results represent a selected group of patients with chronic pain and with severe consequences after the injury.

 Although patients reported high frequencies of symptoms, these are seldom assessed in patients with chronic injury-related pain. The results in the present study agree with Smith-Seemiller et al.; they demonstrated that post-concussion symptoms were common in patients with chronic pain [19]. Since several studies have shown a lack of specificity of PCS [15, 28], the challenge is to establish a causal link between MTBI and PCS and to the diagnosis post-concussion disorder. According to our findings, the optimization of treatment for PCS requires clinicians to assess postconcussion symptoms, to investigate causes for each patient, and to account for factors such as posttraumatic stress and depression.

## Figures and Tables

**Table 1:** Frequency of occurrence of postconcussion symptoms (Rivermead Postconcussion Symptoms Questionnaire).

	All patients (*n* = 86) (%)	Mean	Women (*n* = 59) (%)	Mean	Men (*n* = 27) (%)	Mean	*P*
Headache	73.5	2.25	51.0	2.21	40.72	2.33	n.s.
Dizziness	61.7	1.70	40.0	1.65	34.9	1.81	n.s.
Nausea/vomiting	29.1	1.01	30.0	1.09	16.3	0.85	n.s.
Sleep disturbance	84.9	2.86	42.0	2.84	45.3	2.88	0.024
Fatigue	90.7	3.01	57.0	3.18	53.5	2.67	n.s.
Irritability	66.3	2.11	54.0	2.14	50.0	2.04	n.s.
Feeling depressed	55.8	1.82	41.0	2.02	40.7	1.41	n.s.
Feeling frustrated	55.8	1.93	40.0	2.03	44.5	1.70	0.046
Poor memory	67.1	1.81	51.0	1.90	53.5	1.63	n.s.
Poor concentration	88.2	2.01	50.0	2.21	52.3	1.59	n.s.
Noise sensitivity	59.5	1.52	45.0	1.87	34.9	1.87	n.s.
Blurred vision	55.8	1.05	31.0	0.96	32.6	1.22	0.001
Sensitivity to light	51.2	1.31	41.0	1.35	44.2	1.22	n.s.
Double vision	26.2	0.44	13.0	0.40	16.3	0.52	n.s.
Restlessness	69.0	1.37	35.0	1.37	48.8	1.37	n.s.
Taking longer to think	74.1	1.05	41.0	1.83	48.8	0.85	0.005

Total score (mean)		27.32	28.33		25.15		n.s.

**Table 2:** Proportion of patients reporting symptoms and symptoms scores (%).

Symptom scores	0 (not experienced symptom)	1 (no longer a problem)	2 (mild problem)	3 (moderate problem)	4 (severe problem)	2–4 (problem)
Headache	14.5	16.9	21.7	22.9	24.1	68.7
Dizziness	23.5	14.8	34.6	22.2	4.9	61.7
Nausea/vomiting	44.4	25.9	17.3	8.6	3.7	29.6
Sleep disturbance	7.1	6.0	19.0	29.8	38.1	86.9
Fatigue	4.8	4.8	14.3	36.9	39.3	90.5
Irritability	9.4	23.5	27.1	27.1	12.9	67.1
Feeling depressed	18.8	25.9	18.8	27.1	9.4	
Feeling frustrated	15.3	28.2	15.3	30.6	10.6	55.3
Poor memory	21.2	20.0	23.5	27.1	8.2	58.8
Poor concentration	11.8	21.2	32.9	22.4	11.8	67.1
Noise sensitivity	40.5	22.6	13.1	13.1	10.7	36.9
Blurred vision	42.2	30.1	13.3	9.6	4.8	27.7
Sensitivity to light	30.5	17.1	23.2	18.3	11.0	52.5
Double vision	75.0	10.7	10.7	2.4	1.2	14.3
Restlessness	32.1	27.4	20.2	11.9	8.3	20.4
Taking longer to think	25.9	30.6	16.5	20.0	7.1	43.6
